# Exploring the Relationship Between Antipsychotic Drug Target Genes and Epilepsy: Evidence From Food and Drug Administration Adverse Event Reporting System Database and Mendelian Randomization

**DOI:** 10.1002/brb3.70467

**Published:** 2025-04-02

**Authors:** Ziqian Yin, Zheng Zhan, Youjia Qiu, Menghan Wang, Jinglin Li, Bingyi Song, Zhouqing Chen, Jiang Wu, Zhong Wang

**Affiliations:** ^1^ Department of Neurosurgery & Brain and Nerve Research Laboratory The First Affiliated Hospital of Soochow University Suzhou Jiangsu Province China; ^2^ Department of Otolaryngology Head & Neck Surgery (ENT) The First Affiliated Hospital of Soochow University Suzhou Jiangsu Province China

**Keywords:** Antipsychotic drugs, Epilepsy, FAERS, Mendelian randomization, SMR

## Abstract

**Background:**

The effect of antipsychotic drugs on epilepsy is controversial, and we performed Food and Drug Administration Adverse Event Reporting System (FAERS) data mining and Mendelian Randomization (MR) analyses to clarify the effects of target genes on epilepsy.

**Method:**

We explored antipsychotic‐induced epilepsy AE signals in FAERS. Gene expression was obtained from the eQTLGen consortium and GTEx project. Epilepsy data were obtained from FinnGen and the International League Against Epilepsy (ILAE). MR, Summary‐data‐based Mendelian Randomization (SMR), and colocalization analysis were sequentially performed, and meta‐analysis was performed on genes with significant expression in MR or SMR to assess the causal relationship between them and epilepsy.

**Result:**

Through FAERS database mining, 63 antipsychotics reported 5121 adverse events in epilepsy. MR identified potential causal associations of 14 drug target genes for epilepsy and its subtypes. MCHR1 and SIGMAR1 were still significant for epilepsy after meta‐analysis with no evidence of heterogeneity or pleiotropy. SMR showed that DRD4 and ADRA1D were strongly associated with epilepsy or its subtypes however, neither gene passed the HEIDI test.

**Conclusion:**

Our study indicates that antipsychotic drugs are associated with a high incidence of epilepsy‐related AEs. MR demonstrated a causal relationship between drug targets and epilepsy. Providing new insights for managing epilepsy patients with psychiatric disorders.

## Graphical abstract

Multi‐source drug‐target Mendelian randomization and colocalization analysis integrated with the FAERS database revealed genetic evidence linking antipsychotic drug target genes to epilepsy, validated through meta‐analysis to elucidate underlying therapeutic mechanisms.

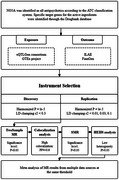

## 1 Introduction

The prevalence of psychiatric disorders among patients with epilepsy is about 5.6% (de Toffol et al. 2018). This clinical complexity introduces significant therapeutic challenges, as certain psychopharmacological agents exhibit proconvulsant properties that may exacerbate seizure activity (Pisani et al. 2002, Kanner and Gidal 2008). Current evidence remains inadequate for delineating precise medication‐specific effects or elucidating genetic interactions influencing seizures. Within the neuropsychiatric management of epilepsy, critical debates persist regarding optimal pharmacotherapeutic strategies and risk mitigation protocols, particularly concerning the delicate balance between psychiatric symptom control and seizure provocation thresholds.

Dopaminergic activity is thought to play an important role in the development of psychiatric disorders, and the clinical efficacy of antipsychotic drugs is strongly correlated with their ability to block subcortical dopamine D2 receptors (Kesby et al. 2018).The study by Bozzi and Borrelli et al. showed that aberrant signaling at the dopamine D1/D2 receptor can induce epilepsy through a dual mechanism: triggering of the PKA‐ERK pathway and activation of the mTOR pathway, with the synergistic action of these two key molecular mechanisms ultimately leading to neuronal cell death, the pathological basis of seizure (Bozzi and Borrelli 2013). In a four‐year observational study by Logothetis et al., all non‐epileptic patients treated with first‐generation antipsychotics had spontaneous seizures (Logothetis 1967). However, the risk varies from drug to drug, and not all antipsychotics induce epilepsy, such as melperone (Gorska et al. 2019, Wolf and Springer Berlin Heidelberg 2004). On the other hand, a study by Pisan et al. suggests that antipsychotic use may contribute to seizures by lowering the seizure threshold (Pisani et al. 2002). Confounding factors, limited by the wide variation in seizure risk, include drug dose, plasma concentration, titration regimen and the patient's hereditary seizure threshold (Pisani et al. 2002). The effect of different antipsychotics on seizure thresholds remains controversial (Bloechliger et al. 2015, Koster et al. 2013, Okazaki et al. 2014, Kumlien and Lundberg 2010, Wang et al. 2024). Retrospective studies cannot avoid confounding factors such as differences in medical standards and sample size. Current studies are not sufficient to clarify the risk of epilepsy caused by all specific antipsychotics, and we performed the present MR analysis with the aim of analyzing the risk of epilepsy caused by antipsychotics at the genetic level.

Mendelian randomization (MR) serves as a statistical tool for establishing causality (Burgess et al. 2023). The method employs genetic information that is closely related to the variable of interest, specifically single nucleotide polymorphisms (SNPs), as instrumental variables. This approach permits the evaluation of causal relationships between exposures and outcomes while circumventing confounding effects and reverse causation concerns. Moreover, MR analysis could also be used to repurpose licensed drugs by integrating summary statistics from genome‐wide association studies (GWAS) and expression quantitative trait locus (eQTL) studies (Interleukin‐6 Receptor Mendelian Randomisation Analysis et al. 2012).

In summary, considering the potential clinical benefits, a comprehensive analysis is needed to explore the relationship and factors influencing antipsychotics and epilepsy‐related adverse events. Based on the reports of antipsychotics in the Food and Drug Administration Adverse Event Reporting System (FAERS) database from 2004 to 2023, epilepsy‐related adverse events were counted, and disproportionality analyses were performed to identify epilepsy adverse events, and drug‐target MR was further used to explore the possible influencing genes of epilepsy that are highly associated with antipsychotic. This study aimed to gain an in‐depth and comprehensive understanding of antipsychotics‐related adverse events in epilepsy and to provide a useful reference for clinical practice.

## 2 Materials and Methods

### 2.1 Identification of Antipsychotic Drugs and Target Genes

We first identified class N05A as all antipsychotics included in this study based on the Anatomical Therapeutic Chemistry (ATC) classification system and obtained the specific active ingredient for each drug under its classification. The target genes of antipsychotic drugs that are widely acknowledged have been identified through the DrugBank database (https://go.drugbank.com/). Detailed information can be found in Table S1.

### 2.2 Epilepsy Adverse Event Data Source and Signal Mining

Different classes of antipsychotics were identified according to the ATC classification system, and the active ingredients, trade names, and aliases of the drugs were searched at Medical Subject Headings (https://www.ncbi.nlm.nih.gov/mesh).We conducted this real‐world, retrospective, observational pharmacovigilance study using the FAERS database. Reported data from the FAERS Publish Dashboard were obtained from the first quarter of 2004 to the fourth quarter of 2023, and data were cleaned with FDA‐recommended de‐emphasis criteria. Epilepsy‐related adverse reactions were coded according to the preferred terminology in the Dictionary of Medical Regulatory Activities. Only medications for which antipsychotic use was primarily suspected were included.

In this study, the ratio of reports (ROR) was used to detect epileptic adverse reaction signals in antipsychotic reports. Seizure adverse reaction signals were considered valid and highly relevant to antipsychotic treatment if there were no fewer than three reports of seizure adverse events and the lower limit of the 95% CI of the ROR exceeded 1. The ROR and 95% CI were calculated as follows: ROR=(a/c)/(b/d); 95% $\mathrm{CI}\;=e^{ln(ROR)\pm 1.96\surd (1/a+1/b+1/c+1/d)}$

### 2.3 Data Sources for MR Analysis

For exposure, we used two sources of publicly available data, selecting data from the eQTLGen consortium to identify drug‐specific target gene expression in blood tissue (https://www.eqtlgen.org/cis‐eqtls.html) (Vosa et al. 2021) and data from the GTEx project to identify drug target gene expression in brain cortex tissue (https://www.gtexportal.org/home/) (Consortium 2020).

The GWAS study of epilepsy and its subtypes (generalized epilepsy, focal epilepsy) was obtained from the International League Against Epilepsy (ILAE) Consortium on Complex Epilepsies in 2018 (International League Against Epilepsy Consortium on Complex 2018) and FinnGen consortiums. The inclusion population for ILAE are as follows: Seizures and epilepsy syndromes were diagnosed according to the ILAE classification. Epileptologists at each participating site evaluated and categorized all cases into subtypes based on electroencephalogram, imaging, and clinical history. Phenotypic categories included hereditary generalized epilepsy (GE), focal epilepsy (FE), and unclassified epilepsy. FinnGen study is a global research program that combines genomic information with digital healthcare data. In Round 9, the endpoints for epilepsy and subtypes were defined using the International Classification of Diseases, Tenth Revision (ICD‐10), and detailed information can be found in Table S2.

### 2.4 MR Design and Instrument Selection

**FIGURE 1:**
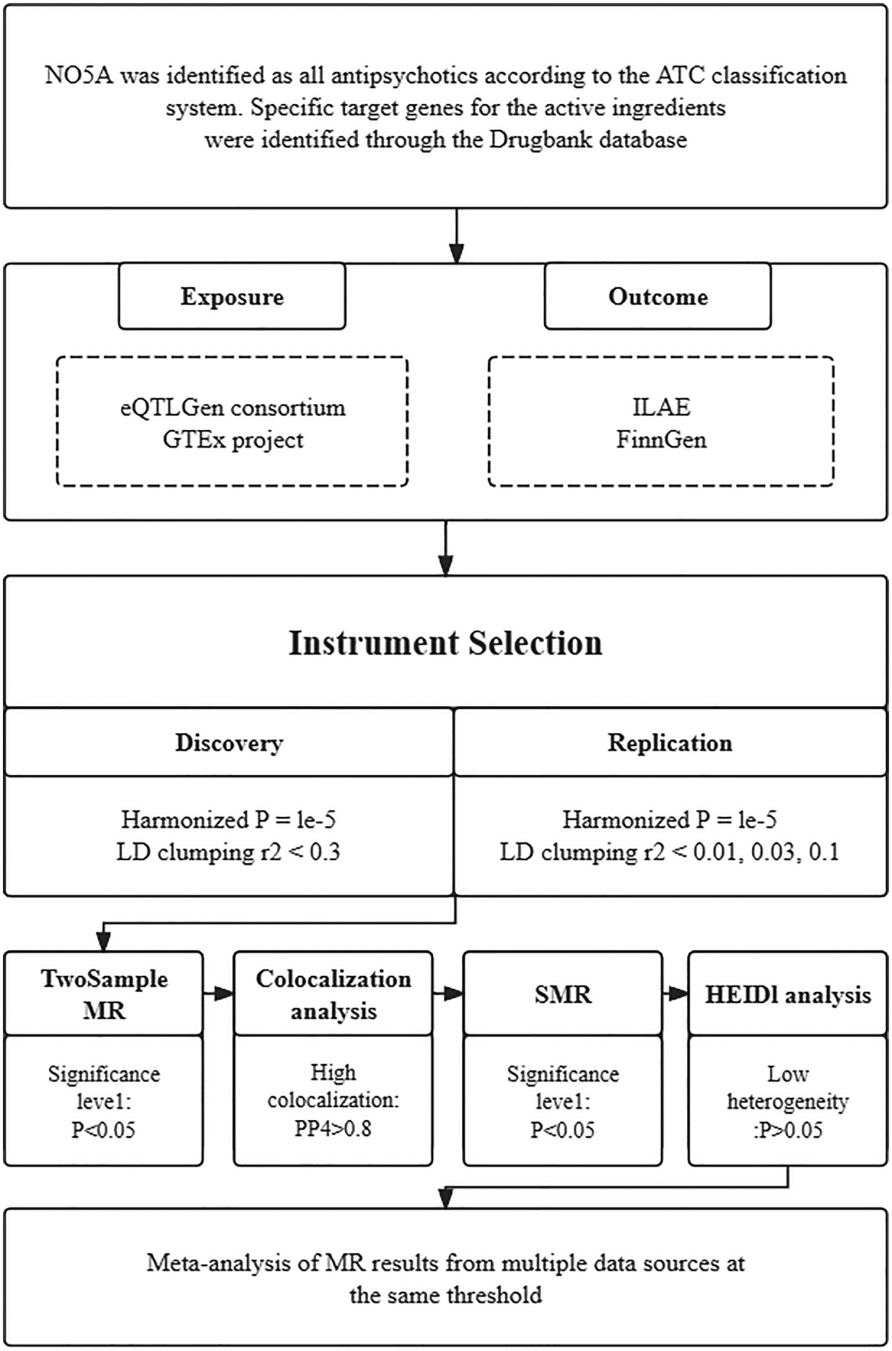
Study design. **ATC,** Anatomical Therapeutic Chemistry; **HEIDI,** Heterogeneity in Dependent Instruments; **ILAE,** International League Against Epilepsy; **LD,** linkage disequilibrium; **MR**: Mendelian Randomization; **PP.H4**: Posterior Probability H4; **SMR**: Summary‐data‐based Mendelian Randomization.

We strictly follow the guidelines outlined in “STROBE‐MR” (Strengthening the Reporting of Observational Studies in Epidemiology‐Mendelian Randomization) (Skrivankova et al. 2021, Hemani et al. 2018). The overall study design is shown in Figure 1. This MR study was required to obey three fundamental assumptions: (1) Genetic instruments should be directly related to the exposure; (2) genetic instruments should not be connected to the outcome and are not associated with the confounding factors; and (3) genetic variants could only affect the risk of outcome through intervening in the exposure. This analysis was conducted in a population of homogenous European ancestry, by which the influence of extraneous variables could be reduced. In addition, the databases of the exposure and outcome were obtained from different sources, which could reduce the possibility of overlapping populations and minimize the potential biases in MR estimates.

Cis‐eQTLs (eQTL located within 1 Mb on either side of the encoding gene) were extracted from GTEx‐V8 (cerebral cortex) and eQTLgen (whole blood) and were then used as IVs to proxy antipsychotic target genes (minor allele frequency > 1%, p1< 1e‐05). Then, we performed linkage disequilibrium (LD) clumping to identify independent SNPs with a threshold of r2< 0.3, kb = 10,000, using the 1000 Genomics Project Phase 3 as the reference panel. During the harmonization of SNP‐exposure and SNP‐outcome effects, we excluded SNPs characterized by mismatched alleles, palindromic sequences, and those harboring missing values. After that, a steiger filter test was performed to ensure that the variants were more strongly associated with the outcome than with the exposure (Hemani et al. 2017). To remove weak instrument bias, SNPs with F‐statistics, calculated using the formula F = beta^2^/se^2^, below the threshold of ten were excluded (Burgess et al. 2011). To enhance the credibility and stability of MR results, we conducted duplicate MR analysis, sensitivity analysis, and consistency test using different aggregation thresholds (r^2^ equal to 0.2, 0.05, and 0.01). In addition, Summary‐data‐based Mendelian Randomization (SMR) analysis was performed by selecting top eQTL SNPs using IVs extracted from these two tissues.

### 2.5 Colocalization and Meta‐analysis

Colocalization analysis was performed for genes with significant results (*p* < 0.05) in the MR analysis to test whether the exposure and the outcome shared the same causal variant. In addition, we performed a meta‐analysis of MR results from multiple data sources at the same aggregation threshold and screening threshold to obtain more robust results. The posterior probabilities generated in the colocalization results are comprised of five hypotheses. H0: The genetic locus under consideration is not associated with the two traits. H1: The locus under consideration is associated with trait 1. H2: The locus is associated with trait 2. H3: The locus is associated with both trait 1 and trait 2, but the association is realized through independent SNPs. H4: The locus is associated with both trait 1 and trait 2, and the association is realized through shared SNPs. Genetic loci are associated with both trait 1 and trait 2 and are realized through shared SNPs (Giambartolomei et al. 2014). Colocalization effects were considered possible when H4 was greater than 50% and highly likely when H4 reached 80% (Zhou et al. 2021).

### 2.6 Statistical Analysis

In this MR analysis, Inverse variance weighted (IVW) was used as the primary analytical method, and MR‐Egger, weighted median, and weighted mode were used to assist in the judgment. When only one instrumental variable was available, we took Wald radio instead of assessment (Boehm and Zhou 2022). The IVW approach provides reliable estimates of exposure‐ and outcome‐related causal effects, provided that the genetic variance meets the three instrumental variable assumptions and that there is no horizontal pleiotropy and no significant heterogeneity. To visualize the MR results, we used odds ratios (OR) and their corresponding 95% confidence intervals (CIs) (Bowden et al. 2015). The Weight Median approach allows for the integration of multiple genetic variation data sets into a single causality estimate, provided that at least 50% of the SNPs are valid (Bowden et al. 2016).

The stability of the MR results was verified through a series of sensitivity analyses, including the Cochran Q test, the MR‐Egger intercept, the leave‐one‐out test, and the funnel plot. A *p*‐value of less than 0.05 for the Cochran Q‐test indicates a significant heterogeneity of results. Meanwhile, the MR‐Egger intercept was employed to ascertain whether the findings were influenced by pleiotropy (Hemani et al. 2018). Furthermore, we applied individual SNP analysis and leave‐one‐out analysis to test the probability of associations identified by individual SNP drivers. For heterogeneity and pleiotropy in SMR, we used the Heterogeneity in Dependent Instruments (HEIDI) test created by Zhu et al. (2016), which specifically tests against the original hypothesis of the existence of a single null variant, which is biologically equivalent to testing for heterogeneity in SNP effect sizes estimated in cis‐eQTL regions of interest.

Analysis was performed using R (version 4.1.0). Software packages used included SMR (version 1.0.9), MendelR (version 1.2.2), and meta (version 6.5‐0).

### 2.7 Standard Protocol Approvals, Registrations, and Patient Consents

The current study was based on a secondary analysis of publicly available databases. Consent was obtained from all participants for the publicly available data, and there was no need to repeat the process of obtaining participant consent and separate institutional review board approval for this study.

## 3 Results

### 3.1 Epilepsy Adverse Event Signals for Antipsychotic Drugs

The ATC code for antipsychotic drugs is N05A, which involves a total of 63 drug active ingredients; the drug name, active ingredient, and alias are listed in the Table S1. After excluding cases with possible epilepsy adverse reactions due to coadministration and/or adverse reactions and related therapeutic indications, a total of 227,700 case‐specific adverse reactions with antipsychotics were reported, of which 5121 epilepsy‐related adverse reactions were reported. Antipsychotic drugs were significantly associated with the occurrence of epilepsy‐related adverse reactions (ROR = 1.672, 95% CI: 1.627 ‐ 1.718, *p* < 0.0001). Detailed adverse event reports with epilepsy are shown in Table 1.

**TABLE 1:** Characteristics of reports with epilepsy related adverse events sourced from the FAERS database (2004 Q1‐2023 Q4).

Variables	No target PT (*N* = 222579)	Target PT (*N* = 5121)	Overall (*N* = 227700)
**Gender**			
Female	100748 (45.3%)	2384 (46.6%)	103132 (45.3%)
Male	93216 (41.9%)	2382 (46.5%)	95598 (42.0%)
Missing	28615 (12.9%)	355 (6.9%)	28970 (12.7%)
**Weight**			
< 50 kg	3996 (1.8%)	156 (3.0%)	4152 (1.8%)
> 100 kg	9525 (4.3%)	247 (4.8%)	9772 (4.3%)
50～100 kg	33330 (15.0%)	1109 (21.7%)	34439 (15.1%)
Missing	175728 (79.0%)	3609 (70.5%)	179337 (78.8%)
**Age**			
< 18	7608 (3.4%)	306 (6.0%)	7914 (3.5%)
Ø 85	2120 (1.0%)	40 (0.8%)	2160 (0.9%)
18～64.9	89918 (40.4%)	2759 (53.9%)	92677 (40.7%)
65～85	15172 (6.8%)	289 (5.6%)	15461 (6.8%)
Missing	107761 (48.4%)	1727 (33.7%)	109488 (48.1%)
**Reporter**			
CN	76147 (34.2%)	1558 (30.4%)	77705 (34.1%)
HP	13629 (6.1%)	210 (4.1%)	13839 (6.1%)
LW	5299 (2.4%)	71 (1.4%)	5370 (2.4%)
MD	62367 (28.0%)	1699 (33.2%)	64066 (28.1%)
OT	31675 (14.2%)	776 (15.2%)	32451 (14.3%)
PH	19239 (8.6%)	337 (6.6%)	19576 (8.6%)
RN	36 (0.0%)	2 (0.0%)	38 (0.0%)
Missing	14187 (6.4%)	468 (9.1%)	14655 (6.4%)
**Year**			
2019	14545 (6.5%)	284 (5.5%)	14829 (6.5%)
2020	13303 (6.0%)	212 (4.1%)	13515 (5.9%)
2021	12225 (5.5%)	180 (3.5%)	12405 (5.4%)
2022	11671 (5.2%)	172 (3.4%)	11843 (5.2%)
2023	12136 (5.5%)	260 (5.1%)	12396 (5.4%)
**Country**			
France	1689 (0.8%)	51 (1.0%)	1740 (0.8%)
Germany	1842 (0.8%)	63 (1.2%)	1905 (0.8%)
Japan	6527 (2.9%)	219 (4.3%)	6746 (3.0%)
UK	3939 (1.8%)	158 (3.1%)	4097 (1.8%)
US	82146 (36.9%)	1176 (23.0%)	83322 (36.6%)
Missing	6455 (2.9%)	307 (6.0%)	6762 (3.0%)

**Abbreviations: CN**, Consumer; **HP**, Health Professional; **LW**, Legal Writer; **MD**, Medical Doctor; **OT**, Other: **PH**, Pharmacist; **PT**, Preferred Term; **RN**, Registered Nurse; **UK,** United Kingdom; **US,** United States.

### 3.2 MR Analysis

**FIGURE 2:**
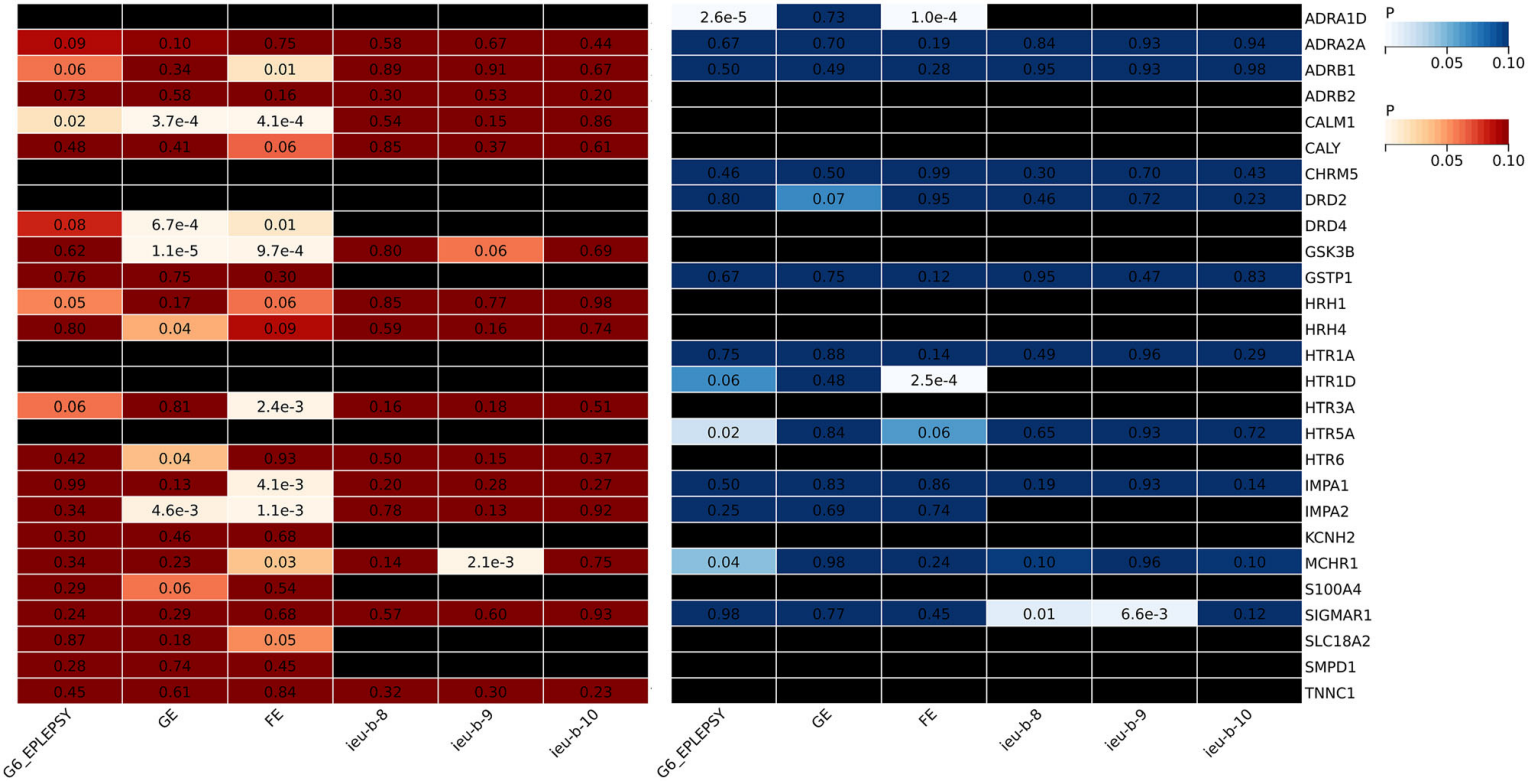
Heatmap of P value from MR analysis results of gene with epilepsy. The red part represents gene expression data from blood samples. The blue part represents gene expression data from the brain cortex tissue. The black part represents no enough snp for MR analysis. **FE**, Focal epilepsy in FinnGen;**G6_EPILEPSY**, Epilepsy in FinnGen; **GE**, Generalized epilepsy in FinnGen; **Ieu‐b‐8**, Epilepsy in ILAE; **Ieu‐b‐9,** Generalized epilepsy in ILAE; **Ieu‐b‐10**, Focal epilepsy in ILAE; **ILAE,** International League Against Epilepsy; **MR**, Mendelian Randomization.

56 target genes were searched for according to the Drugbank database to find relevant target genes (Table S1). We extracted data on the blood tissue expression of 21 drug target genes from the eQTLGen consortium, and data in cerebral cortex tissue expression of 13 drug target genes from the GTEx project. Data of the expression of all genetically predicted genes subjected to the steiger test are presented in Table S3. The P‐value heatmap of the MR analysis is presented in Figure 2.

#### 3.2.1 MR Analysis in Blood Tissue

Expression data for 21 genes were extracted from gene expression data in blood tissues after removal of chain imbalance at r^2^ = 0.3. The results of the MR analysis are presented in Table S4. Using mainly the method of Wald radio and IVW, MR was first performed with epilepsy from FinnGen data, and genetically predicted lower levels of CALM1 were associated with an increased risk of epilepsy. In addition, nine genes (HRH4, HTR3A, HTR6, GSK3B, CALM1, IMPA2, ADRB1, MCHR1, IMPA1) were negatively and five genes (IMPA2, GSK3B, MCHR1, DRD4, CALM1) were positively associated with FE or GE. Analyses with ILAE‐sourced epilepsy yielded that genetically predicted higher levels of MCHR1 were strongly associated with an increased risk of GE. Trends in these associations were generally consistent across other analytical methods, including weighted mode, weighted median, and MR‐Egger. Detailed MR analysis data can be found in Table S4. No heterogeneity or pleiotropy was found (P heterogeneity > 0.05, P pleiotropy > 0.05) (Table S5). A meta‐analysis of epilepsy from both sources suggested that higher levels of MCHR1 were significantly associated with an increased risk of GE (OR: 1.09, 95% CI: 1.04 ‐ 1.16, *p* = 0.001). The scatter plot, of MCHR1 with GE did not show significant heterogeneity. A funnel plot depicted the distribution of SNPs, and a symmetric distribution indicates no horizontal pleiotropy. The leave‐one‐out plot did not identify individual anomalous SNPs leading to a strong driver causal effect. Detailed information was shown in Figures S1‐6. The results remained largely consistent when r^2^ was set at different thresholds.

#### 3.2.2 MR Analysis in Cortex Tissue

In the gene expression data of brain cortex tissues, the expression data of 13 genes were extracted after removing the chain imbalance with the criterion of r = 0.3. The results of the MR analysis are presented in Table S4. MR analyses of epilepsy with FinnGen data, mainly using the methods of Wald radio and IVW, genetically predicted lower levels of HTR5A and HTR1D were associated with an increased risk of epilepsy and FE, respectively. Higher levels of ADRA1Dand MCHR1 were associated with a higher risk of epilepsy. Analyses with epilepsy data from ILAE yielded higher levels of SIGMAR associated with increased risk of epilepsy and GE. Trends in these associations were generally consistent across other analytical methods, including weighted mode, weighted median, and MR‐Egger. Detailed MR analysis data can be found in Table S4. No heterogeneity or pleiotropy was found (P‐heterogeneity > 0.05, P‐pleiotropy > 0.05) (Table S5). After meta‐analysis, the results showed that higher levels of SIGMAR were significantly associated with an increased risk of GE (OR: 1.14, 95% CI: 1.00 ‐ 1.30, *p* = 0.0497). There were not enough SNPs for leave‐one‐out plot, scatter plot and funnel plot. Detailed information was shown in Figures S6‐12. The results remained largely consistent when r^2^ was set at different thresholds.

### 3.3 Colocalization and SMR Analysis

We performed colocalization analyses of gene targets with significant results in MR to further determine the possibility of common causal genetic variants associated with antipsychotic drug target genes and epilepsy and subtypes. The results suggest that DRD4 may have causal variants in FE (PP.H4 > 0.5). Detailed results are presented in Table S7, including regional association maps. We selected genes with significant performance in MR from different GWAS data sources for meta‐analysis, and finally SIGMAR1 and HRH4 remained significant after meta‐analysis (Table 2).

**TABLE 2:** Summary results from MR, meta, colocalization, and SMR for 14 MR‐identified genes.

	Gene	Outcome	MR				Meta		Colocalization		SMR					
			FinnGen		ILAE				FinnGen	ILAE	FinnGen			ILAE		
			Beta	P	Beta	P	OR (95%CI)	P	PP.H4	PP.H4	Beta	P	P HEIDI	Beta	P	P HEIDI
**Blood**	ADRB1	FE	−0.099	0.01	0.018	0.67	0.96(0.86, 1.08)	0.48	1.30%	0.49%	−0.0099	0.95	0.49	−0.0009	0.98	0.42
	CALM1	GE	0.362	< 0.01	−0.017	0.15	1.15(0.75, 1.75)	0.52	7.38%	0.81%	—	—	—	−0.0279	0.76	0.04
	CALM1	FE	−0.165	< 0.01	−0.067	0.86	0.92(0.79, 1.08)	0.30	8.44%	0.54%	—	—	—	0.02339	0.70	0.24
	CALM1	Epilepsy	−0.093	0.02	−0.006	0.54	0.95(0.88, 1.02)	0.18	2.84%	0.45%	—	—	—	−0.0079	0.88	0.56
	GSK3B	GE	−0.214	< 0.01	0.042	0.06	0.92(0.72, 1.18)	0.52	5.00%	0.90%	0.08555	0.71	NA	−0.0026	0.97	0.55
	GSK3B	FE	0.073	< 0.01	−0.005	0.69	1.03(0.96, 1.11)	0.41	1.21%	0.47%	−0.1008	0.32	NA	−0.0051	0.91	0.98
	HRH4	GE	−0.752	0.04	0.164	0.16	0.80(0.33, 1.94)	0.62	3.95%	1.06%	—	—	—	0.02901	0.92	NA
	HTR3A	FE	−0.287	< 0.01	−0.047	0.51	0.85(0.68, 1.08)	0.19	2.24%	0.69%	0.2706	0.38	0.69	0.01023	0.92	0.07
	HTR6	GE	−0.215	0.04	0.057	0.15	0.94(0.72, 1.22)	0.64	3.15%	2.00%	0.03858	0.91	NA	−0.0634	0.50	0.40
	IMPA1	FE	−0.044	< 0.01	−0.012	0.27	0.97(0.94, 1.00)	0.09	0.99%	0.56%	−0.1284	0.08	NA	−0.0051	0.82	0.73
	IMPA2	FE	0.061	< 0.01	0.028	0.92	1.03(0.97, 1.09)	0.32	1.47%	7.08%	0.02542	0.90	NA	0.04177	0.09	0.26
	IMPA2	GE	−0.121	< 0.01	0.001	0.13	0.96(0.83, 1.11)	0.58	3.13%	1.49%	0.26683	0.58	NA	0.01831	0.60	0.65
	MCHR1	GE	0.075	0.23	0.095	< 0.01	1.09(1.04, 1.16)	< 0.01	3.34%	1.67%	—	—	—	−0.1211	0.08	0.09
	MCHR1	FE	−0.065	0.03	0.008	0.75	0.97(0.91, 1.05)	0.47	0.68%	0.46%	—	—	—	−0.0395	0.39	< 0.01
	DRD4	GE	0.330	< 0.01	—	—	—	—	8.96%	46.50%	−0.2959	0.67	0.72	0.24452	< 0.01	< 0.01
	DRD4	FE	0.107	0.01	—	—	—	—	5.37%	1.31%	−0.3371	0.27	0.66	0.05404	0.35	0.09
**Brain cortex**	ADRA1D	Epilepsy	0.109	< 0.01	—	—	—	—	15.50%	0.12%	−0.0979	0.03	NA	—	—	—
	ADRA1D	FE	0.129	< 0.01	—	—	—	—	11.22%	0.46%	−0.1067	0.06	NA	—	—	—
	HTR1D	FE	−0.060	< 0.01	—	—	—	—	4.97%	0.43%	0.06825	0.06	0.83	0.00566	0.74	0.92
	HTR5A	Epilepsy	−0.166	0.02	−0.018	0.65	0.92(0.80, 1.07)	0.27	8.02%	0.36%	—	—	—	—	—	—
	MCHR1	Epilepsy	0.142	0.04	−0.073	0.10	1.03(0.83, 1.27)	0.80	5.17%	2.57%	—	—	—	—	—	—
	SIGMAR1	Epilepsy	−0.002	0.98	0.084	0.01	1.06(0.99, 1.15)	0.11	1.01%	8.87%	—	—	—	—	—	—
	SIGMAR1	GE	−0.058	0.77	0.157	< 0.01	1.14(1.00, 1.30)	< 0.05	2.98%	23.72%	—	—	—	—	—	—

**Abbreviations: FE,** Focal Epilepsy; **GE,** Generalized Epilepsy; **ILAE**: International League Against Epilepsy. **MR,** Mendelian Randomization; NA: not applicable. **SMR**: Summary‐data‐based Mendelian Randomization

## 4 Discussion

To our knowledge, we present the first investigation into the possible links between antipsychotic drugs and epilepsy, utilizing data from the FAERS database and MR analysis. Our initial step involved a comprehensive search of the FAERS database for reports of adverse events related to epilepsy induced by antipsychotic drugs. This search yielded 5,121 instances of epileptic adverse events associated with 63 distinct antipsychotic drugs. Subsequently, we conducted a two‐sample MR analysis. For this, we sourced gene expression data for antipsychotic drug target genes from both blood samples and cerebral cortex tissues. The blood sample data was obtained from the eQTLGen consortium, while the cerebral cortex tissue data was derived from the GTEx project.

In our MR analysis, we identified associations between the expression of 14 genes in blood samples or brain cortex tissues and the risk of epileptic events. We then proceeded with a series of analyses, including meta‐analysis, SMR analysis, and colocalization analysis using gene expression data from multiple sources. After meta‐analysis, we found that elevated expression levels of MCHR1 and SIGMAR1 were significantly correlated with an increased risk of GE. However, the pharmacological implications of these targets remain to be fully understood. Our colocalization analysis did not identify genes that are likely to share common pathogenic variants with epilepsy or its subtypes. Meanwhile, the SMR analysis indicated a strong association between the expression of DRD4 and ADRA1D and epilepsy or its subtypes. However, neither gene passed the HEIDI test, suggesting that the observed associations may not be causal. In conclusion, our study provides valuable insights and a Mendelian reference for the clinical management of psychiatric disorders and epilepsy, potentially informing future treatment strategies.

Patients with psychiatric disorders, particularly those with suicidal tendencies and depression, often suffer from comorbid epilepsy (Tellez‐Zenteno et al. 2007). The management of these patients is highly challenging, and the potential seizure risk associated with antipsychotic drugs remains a persistent subject of concern in clinical practice. Previously available clinical studies on the association between antipsychotic drugs and epilepsy have always been highly controversial and divergent. It is noteworthy that the epileptogenic risk varies significantly across different antipsychotic agents, precluding a uniform risk classification. Alper et al. accessed the FDA public data and conducted a statistical analysis of the incidence of seizures in clinical trials of antipsychotic drugs from 1985 to 2004. They found that the incidence of seizures significantly increased with the use of antipsychotic drugs, with clozapine and olanzapine accounting for the majority (Alper et al. 2007). Conversely, a retrospective observational study conducted by Ribot et al. suggested that antipsychotic drugs, such as selective serotonin reuptake inhibitor or serotonin‐norepinephrine reuptake inhibitors, do not influence the frequency of seizures (Ribot et al. 2017). It is worth noting that there are also reports suggesting that antipsychotic drugs can reduce the frequency of epileptic seizures. For example, it has been reported that the use of therapeutic doses of aripiprazole, mesoridazine, and sulpiride can reduce the occurrence of epileptic seizures in patients with epilepsy (Okazaki et al. 2014, Citraro et al. 2015, Benkert and Hippius 2000).

It should be noted that traditional observational studies often have unavoidable limitations due to confounding factors such as time, ethnicity, and differences in the level of medical care in different regions. RCT's inability to address concerns about long‐term effects. To circumvent potential confounding factors and investigate causal associations. MR presently offers a more expedient and powerful approach to investigating drug targets (Skrivankova et al. 2021). This method, by definition, evaluates the involvement of a particular drug target in relation to the drug itself, rather than the effect of drug use on the outcome. Moreover, it enables the approximation of genetic markers to assess the enduring role of the drug target throughout an individual's lifetime, in contrast to the restricted observation period of traditional observational studies. The aforementioned method has gained significant popularity in assessing the impact of drug targets on various outcomes. For example, Zheng et al. (2022) found a reduction in the risk of Alzheimer's disease through the targeting of metformin drugs. Similarly, Chauquet et al. (2021) found a causal relationship between anti‐hypertensive drug targets and psychiatric disorders.

Melanin‐concentrating hormone is a neuroendocrine factor that has an appetite‐stimulating effect, inhibits energy metabolism, and affects behavior (Shi 2004). It is mainly secreted by the hypothalamus and acts on its receptor, MCHR1, to regulate appetite and energy homeostasis. A study by Stevelink et al. suggests that changes in hormone levels may affect an individual's susceptibility to epileptic seizures (International League Against Epilepsy Consortium on Complex 2023). The meta‐analysis of the findings in this study found that higher levels of MCHR1 are associated with a higher risk of GE. There is limited information regarding human MCH and MCHR1. Previous research on MCHR1 has focused on its functions in human metabolism, and more basic research is needed in the future to explore its role in epileptic seizures. The SIGMAR1 is a widely expressed multifunctional inter‐organelle signaling chaperone protein. Recessive mutations in SIGMAR1 have been identified as causative genes for neuronal and neuromuscular disorders (Aishwarya et al. 2021). In recent years, SIGMAR1 has been considered to be closely related to epileptic seizures and seizure‐related comorbidities (Pong et al. 2022, Riva et al. 2021, Piechal et al. 2021). There is still some controversy about the role of SIGMAR1 in epilepsy. In fact, multiple preclinical and clinical studies have shown that SIGMAR1 exhibits a significant biphasic dose‐response (Maurice 2021). This response is reflected in epilepsy models, where it shows a protective effect at low doses, but at high doses it induces epilepsy by lowering the seizure threshold (Vavers et al. 2023, Payandemehr et al. 2012, Yourick et al. 1999, Tortella et al. 1984). Our research results indicate that high levels of SIGMAR1 expression are associated with a higher risk of GE. This conclusion still needs to be further confirmed in basic research.

The strengths of this study lie in our rigorous approach to precisely defining all compounds classified as antipsychotic drugs using the ATC classification system. We comprehensively searched for the target genes of these drugs using the DrugBank database. Furthermore, we systematically collected expression data for related genes from the eQTLGen consortium and GTEx project. In terms of study outcomes, we selected GWAS data for epilepsy and its subtypes from two different sources and conducted a meta‐analysis to identify genes with potential causal relationships. To enhance the rigor of our results, we set different r^2^ thresholds for data validation. For genes showing potential causal associations, we further conducted SMR and colocalization analyses. Although the functions and mechanisms of some genes are not fully elucidated, our study has confirmed MCHR1 and SIGMAR1 as promising target genes and drug targets for further research. Despite our efforts to maintain rigor, it is important to acknowledge the limitations of this study. Firstly, the data on drug target genes were sourced from the eQTLGen consortium and GTEx project. However, expression data for certain antipsychotic‐associated target genes, such as DRD2 and DRD3, were unavailable in these datasets. While this study provides insights into potential drug‐epilepsy associations, the current evidence does not rigorously support definitive conclusions about specific drugs most likely to induce epilepsy. Future studies incorporating larger sample sizes and more comprehensive eQTL datasets are needed to address this limitation and enhance the reliability of such predictions. The second point is that there may be slight differences in the diagnostic criteria for epilepsy and its subtypes between the Epilepsy Alliance and the Finnish databases, which may have influenced the assessment of the results to some extent. Third, the current investigation utilized European population‐derived eQTL and GWAS data. Due to inherent limitations in the original datasets, we were unable to conduct comprehensive stratified analyses across diverse populations, genders, or other demographic variables. Future investigations should prioritize examining potential variations across racial groups, gender, and other demographic strata. Fourth, the FAERS database relies on voluntary patient reporting, which may result in data accuracy issues such as misreporting and underreporting; therefore, it cannot achieve precise quantification of antipsychotic‐related seizure risks.

## 5 Conclusion

In conclusion, our study demonstrated that antipsychotic drug use is often accompanied by a higher incidence of epilepsy‐related adverse events. MR analysis identified 14 drug target genes causally associated with epilepsy or its subtypes and identified MCHR1 and SIGMAR1 as drug target genes of interest for future studies in epilepsy. To strengthen and validate the results of this analysis, future investigations should include targeted large‐sample RCTs.

## Author Contributions

ZQY and ZZ provided the original manuscript, and ZW and JW provided the ideas for this article. ZQY and ZZ carried out the data analysis and table drawing for this article. YJQ embellished and revised the article. MHW and BYS searched and screened the data sources.

## Conflicts of Interest

The authors declare no conflicts of interest.

### Peer Review

The peer review history for this article is available at https://publons.com/publon/10.1002/brb3.70467.

## Institutional Review Board Statement

The current study was based on a secondary analysis of publicly available databases. Consent was obtained from all participants for the publicly available data, and there was no need to repeat the process of obtaining participant consent and separate institutional review board approval for this study.

## Standard Protocol Approvals and Patient Consents

The current study was based on a secondary analysis of publicly available databases. Consent was obtained from all participants for the publicly available data, and there was no need to repeat the process of obtaining participant consent and separate institutional review board approval.

## Supporting information



Supplementary Materials.

Supplementary Materials.

## Data Availability

The data enrolled in the study could be found in the manuscript and the supplementary materials.
