# Supplementary material for: Exploring the Relationship Between Antipsychotic Drug Target Genes and Epilepsy: Evidence From Food and Drug Administration Adverse Event Reporting System Database and Mendelian Randomization
Source: Brain Behav. 2025 Apr 2;15(4):e70467. doi: 10.1002/brb3.70467 (PMC11962216; doi:10.1002/brb3.70467)
Supplement: Supplementary file 1 — Supplementary Materials. [file BRB3-15-e70467-s001.DOCX]

**Exploring the Relationship Between Antipsychotic Drug Target Genes and Epilepsy: Evidence from FAERS database and Mendelian Randomization**

Ziqian Yin^1,†^, Zheng Zhan^1,†^, Youjia Qiu^1,†^, Menghan Wang^1^,Jinglin Li^1^,Bingyi Song^1^, Zhouqing Chen^1,*^, Jiang Wu^1,*^, Zhong Wang^1,*^

^1^Department of Neurosurgery & Brain and Nerve Research Laboratory, The First Affiliated Hospital of Soochow University, Suzhou, Jiangsu Province, China

^2^ Department of Ent, The First Affiliated Hospital of Soochow University, Suzhou, Jiangsu Province, China

^†^Ziqian Yin, Zheng Zhan, and Youjia Qiu contributed equally to this work.

*** Correspondence:**

Jiang Wu, Department of Neurosurgery, The First Affiliated Hospital of Soochow University, 188 Shizi Street, Suzhou, Jiangsu Province, 215006, China. Email address: [sdfyywujiang@163.com](mailto:sdfyywujiang@163.com).

Zhouqing Chen, Department of Neurosurgery, The First Affiliated Hospital of Soochow University, 188 Shizi Street, Suzhou, Jiangsu Province, 215006, China. Email address: [zqchen6@163.com](mailto:zqchen6@163.com).

Zhong Wang, Department of Neurosurgery, The First Affiliated Hospital of Soochow University, 188 Shizi Street, Suzhou, Jiangsu Province, 215006, China. Email address: [wangz8761@163.com](mailto:wangz8761@163.com).

Figure S1: Scatter plot of MCHR1 in blood with GE in FinnGen.

Figure S2: Leave one out plot of MCHR1 in blood with GE in FinnGen.

Figure S3 Funnel plot of MCHR1 in blood with GE in FinnGen.

Figure S4: Scatter plot of MCHR1 in blood with GE in ILAE.

Figure S5: Leave one out plot of MCHR1 in blood with GE in ILAE.

Figure S6: Funnel plot of MCHR1 in blood with GE in ILAE.

Figure S7: Scatter plot of SIGMAR1 in brain cortex with FE in FinnGen.

Figure S8: Leave one out plot of SIGMAR1 in brain cortex with FE in FinnGen.

Figure S9: Funnel plot of SIGMAR1 in brain cortex with FE in FinnGen.

Figure S10: Scatter plot of SIGMAR1 in brain cortex with FE in ILAE.

Figure S11: Leave one out plot of SIGMAR1 in brain cortex with FE in ILAE.

Figure S12: Funnel plot of SIGMAR1 in brain cortex with FE in ILAE.

Figure S1: Scatter plot of MCHR1 with GE in FinnGen.

-0.4

0.0

0.4

0.1

0.2

0.3

0.4

0.5

SNP effect on exposure

SNP effect on outcome

MR Test

Inverse variance weighted

MR Egger

Weighted median

Weighted mode

Figure S2: Leave one out plot of MCHR1 with GE in FinnGen.

All

rs133075

rs133067

rs9306345

rs5758024

rs112169312

rs9611478

rs183365469

rs117381839

rs73167017

rs75843224

rs5751038

rs11704314

rs144203347

rs2413631

rs117407521

rs140029666

rs78463154

rs76164940

rs117542783

rs149273008

rs79376201

rs117776121

rs139489

rs141418066

rs183748472

rs12483888

rs9607683

rs4821944

rs4822017

rs35898643

rs5995843

rs76653253

rs12169622

rs5757946

rs11090023

rs9623290

rs3087592

rs116894924

rs78401207

rs118166233

rs144145667

rs147997002

rs76204748

rs73165024

rs117674694

rs113077652

rs137952

rs150221573

rs114607

rs77582394

rs62236841

rs76075068

rs3021227

rs139531

rs77698202

rs73176684

rs56211152

rs56361238

rs113795690

rs78985466

rs5751022

rs7291793

0.0

0.1

0.2

MR leave-one-out sensitivity analysis for

'exposure' on 'outcome'

Figure S3 Funnel plot of MCHR1 with GE in FinnGen.

2

4

6

-2

-1

0

1

β

I

V

1

S

E

I

V

MR Method

Inverse variance weighted

MR Egger

Figure S4: Scatter plot of MCHR1 with GE in ILAE.

-0.05

0.00

0.05

0.1

0.2

SNP effect on exposure

SNP effect on outcome

MR Test

Inverse variance weighted

MR Egger

Weighted median

Weighted mode

Figure S5: Leave one out plot of MCHR1 with GE in ILAE.

All

rs11090023

rs78463154

rs7291793

rs139531

rs9306345

rs137952

rs9607683

rs5995843

rs5751022

rs5751038

rs2413631

rs56361238

rs133075

0.00

0.05

0.10

0.15

MR leave-one-out sensitivity analysis for

'exposure' on 'outcome'

Figure S6: Funnel plot of MCHR1 with GE in ILAE.

5

10

15

20

-0.2

0.0

0.2

0.4

β

I

V

1

S

E

I

V

MR Method

Inverse variance weighted

MR Egger

Figure S7: Scatter plot of SIGMAR1 with FE in FinnGen.

Insufficient number of SNPs

Figure S8: Leave one out plot of SIGMAR1 with FE in FinnGen.

Insufficient number of SNPs

Figure S9: Funnel plot of SIGMAR1 with FE in FinnGen.

Insufficient number of SNPs

Figure S10: Scatter plot of SIGMAR1 with FE in ILAE.

Insufficient number of SNPs

Figure S11: Leave one out plot of SIGMAR1 with FE in ILAE.

Insufficient number of SNPs

Figure S12: Funnel plot of SIGMAR1 with FE in ILAE.

Insufficient number of SNPs
